# Green Tea Extract Attenuates Oxidative Stress and Prolongs Red Blood Cell Survival in Iron-Overloaded β-Thalassemic Mice

**DOI:** 10.3390/antiox15080990

**Published:** 2026-08-10

**Authors:** Yanping Zhong, Sakaewan Ounjaijean, Sirichai Srichairatanakool, Narisara Paradee, Suphatta Yubo, Honghong Xu, Pimpisid Koonyosying, Phatchariya Phannasil, Saovaros Svasti, Somdet Srichairatanakool

**Affiliations:** 1 Department of Biochemistry, Faculty of Medicine, Chiang Mai University, Chiang Mai 50200, Thailand; yanping_z@cmu.ac.th (Y.Z.); narisara.p@cmu.ac.th (N.P.); supatta0210@gmail.com (S.Y.); pimpisid.k@cmu.ac.th (P.K.); 2 School of Medical Technology and Artificial Intelligence, Youjiang Medical University for Nationalities, Baise 533000, China; 3 Research Institute for Health Sciences, Chiang Mai University, Chiang Mai 50200, Thailand; sakaewan.o@cmu.ac.th; 4 Division of Hematology, Department of Internal Medicine, Faculty of Medicine, Chiang Mai University, Chiang Mai 50200, Thailand; sirichai.srichai@cum.ac.th; 5 Department of Biochemistry, Faculty of Medicine, Youjiang Medical University for Nationalities, Baise 533000, China; honghongxu@ymun.edu.cn; 6 Thalassemia Research Center, Institute of Molecular Biosciences, Mahidol University, Salaya, Nakhon Pathom 73170, Thailand; phatchariya.pha@mahidol.ac.th (P.P.); saovaros.sva@mahidol.ac.th (S.S.)

**Keywords:** tea, *Camellia sinensis*, polyphenol, thalassemia, iron overload, red blood cell, antioxidant

## Abstract

Green tea extract (GTE) contains catechin polyphenols with potent antioxidant and iron-chelating activities. This study investigated whether GTE could attenuate oxidative stress and improve red blood cell (RBC) survival in iron-overloaded β-thalassemic mice. Catechin composition and antioxidant activity of GTE and black tea extract (BTE) were evaluated using HPLC-photodiode array detection and ABTS radical scavenging assays. Anti-hemolytic effects were examined in RBC from healthy subjects and transfusion-dependent β-thalassemia patients. In vivo studies were performed using male C57BL/6J wild-type (WT) and heterozygous β-globin knockout (BKO) mice fed a ferrocene-enriched diet to induce iron overload. Mice subsequently received oral GTE (300 mg/kg/day) or deferiprone (DFP, 50 mg/kg/day) for 12 weeks. GTE contained markedly higher levels of catechin derivatives, particularly epigallocatechin-3-gallate, and exhibited stronger antioxidant activity than BTE. GTE significantly reduced RBC hemolysis, reactive oxygen species, malondialdehyde, and plasma non-transferrin-bound iron levels while restoring glutathione concentrations and prolonging RBC survival in iron-overloaded mice. The protective effects of GTE were comparable to DFP treatment. These findings suggest that GTE may serve as a promising natural adjunctive strategy for reducing oxidative injuries in β-thalassemia and iron-overload disorders.

## 1. Introduction

β-Thalassemia comprises a heterogeneous group of inherited hemoglobin disorders caused by reduced or absent β-globin chain synthesis, resulting in excess unpaired α-globin chains, ineffective erythropoiesis, chronic hemolytic anemia, and progressive iron overload [1,2]. The precipitation and oxidation of excess α-globin chains can damage erythroid precursors and mature RBCs, thereby promoting membrane instability and reducing deformability, while resulting in premature clearance and shortened RBC survival [1,3]. In transfusion-dependent thalassemia (TDT) cases, repeated blood transfusions add substantial iron, while ineffective erythropoiesis suppresses hepcidin and increases intestinal iron absorption, further aggravating systemic iron loading [1,4].

When transferrin-binding capacity is exceeded, circulating non-transferrin-bound iron (NTBI) begins to appear. NTBI includes redox-active, chelatable iron species that can enter tissues and catalyze reactive oxygen species (ROS) formation, thereby promoting lipid peroxidation, protein oxidation, membrane damage, and organ injury [5,6]. Iron chelation therapy remains the standard treatment for the management of iron overload in transfusion-dependent β-thalassemia patients. Clinically available chelators, such as DFP, desferrioxamine, and deferasirox, effectively reduce body iron burden and improve survival outcomes [3,7,8]. However, these therapies have been associated with various limitations including high treatment costs, adverse effects, and poor long-term compliance [8]. Therefore, increasing amounts of attention have been focused on naturally derived compounds that possess antioxidant and iron-chelating activities, which may ultimately provide supportive benefits alongside conventional chelation therapy.

Green tea (*Camellia sinensis*) is abundant with catechin polyphenols, particularly epigallocatechin-3-gallate (EGCG), epicatechin (EC), epigallocatechin (EGC), and epicatechin gallate (ECG) [9]. Experimental evidence indicates that EGCG can suppress iron-induced ROS generation, lipid peroxidation, and ferroptotic injury, whereas limited clinical evidence suggests that green-tea consumption may influence indices of iron overload in thalassemia patients [10,11]. However, their effects on RBC survival and susceptibility to oxidative hemolysis, two key pathological features contributing to anemia in β-thalassemia, remain incompletely understood [3,12,13,14,15,16,17,18]. Moreover, few studies have integrated ex vivo evaluation of anti-hemolytic activity in RBC obtained from transfusion-dependent β-thalassemia patients with in vivo assessment of RBC survival under chronic iron-overload conditions. Therefore, the present study investigated the phytochemical composition, antioxidant activity, and anti-hemolytic effects of GTE and BTE in human β-thalassemia RBC. In addition, this study evaluated whether chronic GTE administration could attenuate oxidative stress, reduce plasma NTBI levels, and prolong RBC survival among iron-overloaded BKO thalassemic mice. These findings may provide further insight into the antioxidant and RBC-protective effects of GTE and support its further investigation as a potential adjunctive strategy for mitigating oxidative stress associated with β-thalassemia and iron overload.

## 2. Materials and Methods

### 2.1. Chemicals and Reagents

Aluminum chloride anhydrous powder (Product number 563919, 99.99% pure), L-Ascorbic acid (AA) (Product number: A92902, ≥99% pure), 2,2′-azobis(2-amidinopropane) dihydrochloride (AAPH) (Product number: 440914, 97% pure), butylated hydroxytoluene (BHT) (Product number: PHR1117, 99% pure), deferiprone (DFP) (Product number: 379409, 98% pure), ferrocene (Product number: F408, 98% pure), ferric ammonium citrate (FAC, Product number: RES20400-A7), 4-(2-hydroxyethyl)-1-piperazineethanesulfonic acid) (HEPES) buffer pH 7.4 (Product number: H3375), nitrilotriacetate disodium (Product number: N0128), nitrilotriacetate trisodium (Product number: N0253), phosphate-buffered saline (PBS) pH 7.4 (Product number: P7059), 1,1,3,3-tetramethoxypropane (TMP) (Product number: 108383, 99% pure), and 2-thiobarbituric acid (TBA) (Product number: T5500, ≥98% pure) were purchased from Sigma-Aldrich Chemicals Company Limited (Saint Louis, MO, USA). Authentic standards, such as gallic acid (GA) (Product number: G7384, 97.5% pure), gallocatechin 3-gallate (GCG) (Product number: G6782, ≥99% pure), EGC (Product number: E4143, ≥95% pure), catechin (C) (Product number: C0567, ≥97% pure), EGCG (Product number: E4143, >95% pure), ECG (Product number: E3893, 98% pure), caffeine (CF) (Product number: C1778, 99% pure), and quercetin (Q) (Product number: Q4591, 95% pure), were obtained from the Sigma-Aldrich Chemicals Company. Ellman’s reagent containing 5,5-dithio-bis (2-nitrobenzoic acid) (DTNB) for colorimetric glutathione assay (Catalogue number E-BC-K030-S) was obtained from Wuhan Elabscience Biotechnology Company Limited, Wuhan, China. Dimethyl sulfoxide (DMSO) (Product number 102952), Deionized water (DI), with a density of 0.9985 g/L and a conductivity value of ≤4.3 µS/cm, and all HPLC-grade solvents were purchased from Merck-Millipore Group, Merck KGaA, Darmstadt, Germany. Asian rodent chow (CP082) was purchased from Charoen Pokphand Group Company Limited, Huai Khwang, Bangkok, Thailand. Accordingly, 1-Methyl-2-propyl-3-hydroxypyridine-4-one (CP22) was kindly obtained from Professor Robert C. Hider, PhD., Institute of Pharmaceutical Sciences, King’s College London, London, United Kingdom.

### 2.2. Ethical Approval and Statement

All animal experiments were conducted with approval from the Institutional Animal Care and Use Committee, Faculty of Medicine, Chiang Mai University (Reference No. 03/2548; approved on 23 June 2005). In addition, the study protocol involving human participants was reviewed and approved by the Research Ethics Committee (REC), Faculty of Medicine, Chiang Mai University, Chiang Mai, Thailand (Research ID: 0509; Study Code: BIO-2568-0509; approved on 5 September 2025). The study was conducted in accordance with the Declaration of Helsinki and relevant institutional guidelines. Written informed consent was obtained from all healthy volunteers and patients prior to blood collection. Before initiating this study, a written study protocol describing the research objectives, experimental procedures, and statistical analysis plan was developed and approved by the investigators and the institutional ethics committee. Because this was a preclinical animal study with an ex vivo human laboratory component rather than a clinical trial, the protocol was not registered in a public trial registry.

### 2.3. Tea Samples and Extract Preparations

Fresh tea (*Camellia sinensis*) leaves were harvested and immediately dried in a microwave oven (800 W, 3 min, without lid) [19]. The dried tea leaves were ground and extracted with hot deionized water (80 °C) for 15 min. The extract was filtered through a 0.45 µm cellulose acetate membrane filter (Millipore, Maidstone, UK). The filtrate was freeze-dried to obtain green tea extract (GTE) powder, which was stored in aluminum foil-wrapped plastic containers at −20 °C until use. Black tea leaves were purchased from a supermarket in Baise, Guangxi, China and were manufactured according to the factory’s standard procedures.

### 2.4. Determination of Total Phenolic Content (TPC)

GTE (100 µL) was mixed with 10% (*v*/*v*) Folin–Ciocalteu reagent (200 µL) and 10% (*w*/*v*) sodium carbonate (800 µL), incubated at 25 °C for 30 min, and OD was measured at a wavelength of 700 nm against a reagent blank using a double-beam spectrophotometer. TPC was determined from a standard curve of GA, which was then expressed as mg gallic acid equivalent (GAE)/g [20].

### 2.5. HPLC Quantification of Major Catechins

The catechin derivatives in GTE were quantified using reverse-phase high-performance liquid chromatography coupled with photodiode array detection (HPLC-PDA) according to previously described methods [14,21]. Briefly, 10 µL of GTE solution (1.1 mg) was injected into an HPLC system (i-Series LC-2050, Shimadzu Corporation, Kyoto, Japan) equipped with a PDA detector (SPD-M20A, Shimadzu Corporation). Separation was then achieved using a Waters SpheroSorb ODS2 column (250 × 4.7 mm, 5 µm particle size) with isocratic elution involving a mobile phase consisting of 0.05% H_2_SO_4_:acetonitrile:ethyl acetate (86:12:2, *v*/*v*/*v*) at a flow rate of 1.0 mL/minute. Optical density (OD) was monitored at 210 nm. Analytical data were processed using Shimadzu LabSolutions DB/CS software (version 6.106). Catechin concentrations were quantified by comparison with authentic standards. Chromatographic peaks in the samples were identified by comparing their retention times and the UV spectrum values with those of the reference standards. An equal volume of working standard solution was also injected into the HPLC, and peak area (PA) responses were obtained. External calibration curves were prepared using authentic standards of GA, C, EC, EGC, EGCG, ECG, and GCG. Calibration curves exhibited excellent linearity. Compound concentrations were calculated from peak areas using external standard calibration and expressed as mg/g dry extract. This was established by contrasting integrated PA values of the sample and those of the corresponding standard graph. The limits of detection (LOD) for GA, GCG, EGC, C, EGCG, ECG, and CF were 1.60, 2.28, 1.37, 2.21, 8.31, 0.55, and 20.36 mg/g, respectively.

### 2.6. Determination of Antioxidant Activity

The antioxidant capacity of GTE was evaluated using the ABTS radical cation decolorization assay [22]. The ABTS radical (ABTS^•+^) was generated by reacting 7 mM ABTS with 2.45 mM potassium persulfate, and the resulting solution was diluted with PBS pH 7.4 solution to obtain an OD of 0.70 at 734 nm. In the assay, 20 µL of samples (GTE and BTE), or Trolox standard at concentrations in a range of 0.0156–1 mg/mL, along with PBS were mixed with 1.0 mL of the ABTS^•+^ solution. After incubation at room temperature for 6 min, OD was recorded at 734 nm against a reagent blank using the double-beam spectrophotometer. Percent inhibition of ABTS^•+^ generation was determined using the following Equation (1):

% Inhibition of ABTS^•+^ generation = [(OD_PBS_ − OD_sample_)/OD_PBS_] × 100 (1)

where OD_PBS_ = ABTS^•+^ solution treated with PBS and OD_sample_ = ABTS^•+^ solution treated with Trolox, GTE, or BTE. An inhibitory curve of ABTS^•+^ generation was plotted by contrasting the compound concentrations (*x*-axis) against the percentages of the inhibition (*y*-axis). Half-maximal inhibitory concentration (IC_50_) values of the ABTS^•+^ generation were then determined from the curves.

### 2.7. Study for Effects of Tea Extracts on Normal and Thalassemia RBC Hemolysis

#### 2.7.1. Selection of Subjects

This study selected healthy subjects (*n* = 5), while transfusion-dependent β-thalassemia (TDT) patients (*n* = 14) included β-thalassemia major (bTM) (*n* = 2) and β-thalassemia hemoglobin E (bTE) (*n* = 12) patients who were hospitalized at the Adult Thalassemia Clinic, Maharaj Nakorn Chiang Mai Hospital, Faculty of Medicine, Chiang Mai University, Chiang Mai, Thailand. The primary objective of this ex vivo study was to investigate the protective effects of tea extracts in erythrocytes obtained from transfusion-dependent β-thalassemia patients. Therefore, a larger number of patients (*n* = 14) was recruited to better represent the biological variability of the disease. Healthy volunteers (*n* = 5) served as a reference group to establish the baseline responses of normal erythrocytes to oxidative stress and antioxidant treatment. The subjects were informed of the study objectives and protocol, as well as the advantages and disadvantages of participation. They then submitted their written informed consent. The inclusion criteria identified male/female subjects aged 20–45 years with clinical and laboratory diagnoses confirming β-thalassemia. Additionally, their medication history and vital signs were reviewed while the following were also confirmed: plasma ferritin >1500 ng/mL; no infections; no fever; physician’s recommendation for participation; and voluntary consent and signed informed consent forms. Exclusion criteria eliminated subjects associated with pregnancy or breastfeeding, regular alcohol consumption and substance abuse, alcohol consumption within 48 h prior to the study, regular smoking (>10 cigarettes/day), moderate smoking (<10 cigarettes/day) but who were unable to quit before and during the study, prior participation in other clinical studies, serious infectious diseases, any degree of renal impairment, and any other history deemed inappropriate by a physician for participation. Subject withdrawal criteria included adverse reactions deemed necessary by a physician to warrant withdrawal from the study, failure to comply with the study requirements, and a need to withdraw from the study. Patients meeting the eligibility criteria underwent blood collection [23].

#### 2.7.2. Blood Collection and Preparation

All participants were non-smokers. Before blood collection, participants abstained from alcohol consumption and withheld iron chelating medications for at least 72 h. Subject’s venous blood (15 mL) was collected into lithium heparin-anticoagulant vacutainer tubes, mixed gently, and centrifuged at 1500 *g*, 4 °C for 20 min to separate RBC from the plasma. Afterwards, RBC were washed three times with PBS solution and resuspended in the PBS to make a 10% (*v*/*v*) RBC suspension.

#### 2.7.3. Evaluation of the Protective Effects of Tea Extracts Against Oxidative Hemolysis in Human RBC

The concentration of GTE (1.25 mg/mL) was selected based on preliminary dose-optimization experiments and the outcomes of our previous studies demonstrating consistent protection against iron/H_2_O_2_-induced oxidative injury. This concentration was considered appropriate for evaluating antioxidant efficacy under the more severe oxidative conditions generated by the iron/H_2_O_2_ system. Briefly, 100 μL of GTE, BTE combined with AA (1 mg/mL), and PBS were incubated with human RBC suspension (100 µL) and 200 mM AAPH solution (200 µL) at 37 °C for 2 h in a shaker incubator. The reaction mixtures were then diluted with PBS (400 µL) and centrifuged at 3000× *g* for 10 min. Finally, OD values of the supernatant were measured at 540 nm using a 96-well microplate reader [17]. Percentage hemolysis was calculated using the following Equation (2):

% RBC hemolysis = [(OD_sample_ − OD_PBS_)/(OD_DI_ − OD_PBS_)] × 100 (2)

wherein OD_sample_ = RBC treated with GTE, BTE, or AA, OD_PBS_ = RBC in PBS, and OD_DI_ = RBC in DI.

### 2.8. Effects of GTE Treatment on RBC Survival and Oxidative Stress in Iron-Overloaded Thalassemic Mice

#### 2.8.1. Animal Care

Adult C57BL/6J male mice (initial age 75–120 days), including wild type (WT) (^mu^β^+/+^) and heterozygous β-knock-out thalassemia (BKO) (^mu^β^th−3/+^), were provided by the Thalassemia Research Center, Institute of Molecular Biosciences, Mahidol University, Salaya Campus, Nakorn Pathom Province, Thailand. BKO thalassemic mice were generated by a gene targeting technique according to Jamsai and colleagues [24]. Hematological data of BKO mice resembled human thalassemia intermedia patients with the transfusion-dependent phenotype [25]. Animals were monitored daily throughout the experimental period for general appearance, body weight, activity, grooming behavior, and signs of distress or illness. Humane endpoints included severe lethargy, inability to access food or water, rapid weight loss or severe physical deterioration. No animals reached humane endpoint criteria during the study. No unexpected adverse events were observed.

#### 2.8.2. Protective Effect of GTE Against Oxidative Hemolysis of Mouse RBC

Mice were maintained under approved deep terminal anesthesia following the administration of phenobarbital (60 mg/mL) in accordance with Institutional Animal Welfare and Ethical Review Protocols. Cardiac blood was collected into lithium heparin-coated tubes by well-trained personnel according to experimental requirements and animal welfare guidance. Blood samples were promptly centrifuged at 1500× *g*, 4 °C for 10 min to separate RBC from plasma. The RBC were then washed twice with PBS pH 7.4 solution and prepared in 5% RBC suspension (5% hematocrit). In the assay, the RBC suspension was pretreated with 50 μL of PBS, GTE of DFP at 37 °C for 30 min that had been incubated with 100 μM FAC solution (100 μL), and 3% H_2_O_2_ (100 μL) at 37 °C with gentle shaking for 3 h. Afterwards, the reaction mixture was diluted with 8 mL of the PBS solution, centrifuged at 2000× *g* for 10 min, and the OD was then measured at 540 nm against PBS solution. The percentage of RBC hemolysis was calculated using the following Equation (1):

% RBC hemolysis = (OD_sample_ − OD_PBS_)/(OD_DI_ − OD_PBS_) × 100 (3)

wherein OD_sample_ = RBC treated with GTE of DFP, OD_PBS_ = RBC in PBS, and OD_DI_ = RBC in DI.

#### 2.8.3. Iron Loading and GTE Treatment

Iron-enriched diet (Fe diet) was prepared by adding 0.2% (*w*/*w*) ferrocene to CP082 chow (N diet), wherein 1 kg contained 20–24% protein, 4–5% fat, 50–60% carbohydrate, plant fiber supplemented with vitamin A, D_3_, E, K_3_, B_2_, 180 mg iron, copper, manganese, zinc, and iodine, ultimately yielding 780 mg Fe/kg. The diet was stored in sealed plastic bags in a −20 °C freezer until being used. The GTE dose was selected based on previous studies demonstrating antioxidant and iron-chelating efficacy without observable toxicity in murine models, whereas the DFP dose represents a widely used pharmacologically effective positive-control dose for experimental iron overload [26,27]. WT and BKO mice were fed with the Fe diet for 5 days/week to induce iron overload until 12 weeks. Afterwards, the mice were randomly divided into 4 subgroups (12 mice each) and orally administered with DI and 300 mg GTE (300 mg/kg body weight (BW)/day, which was equivalent to 68.55 mg phenolic content, and DFP (50 mg/kg BW/day along with the Fe diet feeding for another 12 weeks [28]. Blood was withdrawn from the tail veins monthly and the cardiac left ventricle after termination at week 12. The blood samples were then collected in lithium heparin-coated tubes. RBC were separated from plasma by centrifugation as has been described above, and the plasma was kept frozen at −20 °C for further analysis.

#### 2.8.4. Detection of RBC Survival

Three male mice were randomly taken from each group after iron loading and GTE treatment for 2 months. Each mouse received a tail vein injection of 200 µL of 15 mg/mL EZ-Link Sulfo-NHS-Biotin (Pierce, Rockford, IL, USA). A 2–3 µL blood sample was collected at different time periods via the tail vein puncture. RBC were washed in 10 mM HEPES buffer, pH 7.4, containing 165 mM NaCl (HBSM), and the number of biotinylated RBC was determined by incubation of the RBC suspension (5 × 10^6^ cells/mL) with 5 μg/mL phycoerythrin (PE)-conjugated streptavidin (Becton Dickinson Bioscience, San Diego, CA, USA) in HBSM buffer containing 2.5 mM CaCl_2_. Fluorescence intensity (FI) of PE-labelled RBC was measured at an excitation wavelength of 488 nm and an emission wavelength of 575 nm using a BD FACScan flow cytometer (Becton Dickinson Bioscience, San Diego, CA, USA). Data were harvested and analyzed using Cell Quest software version 4.1 (BD Bioscience, San Diego, CA, USA) as has been described earlier [18,29]. An RBC survival curve was generated for determination of the duration that biotinylated RBC were reduced to 50% survival half-time (T_1/2_).

#### 2.8.5. Measurement of ROS

RBC suspension (10^6^ cells/mL) was achieved by incubating RBC with 0.4 mM DCFH-DA at 37 °C for 15 min in a humidified 5% CO_2_ incubator. The FI was then measured at excitation/emission wavelengths of 495 nm/530 nm using the BD FACScan flow cytometer [30]. Data were harvested and analyzed using CellQuest software version 5.0.1. Cell populations were gated based on forward- and side-scatter characteristics to exclude debris. FI was determined from gated populations when compared with the unstained control.

#### 2.8.6. Measurement of Glutathione Content

Reduced glutathione (GSH) concentration was determined using Ellman’s reagent by following the procedure described by Moron et al. [31]. Briefly, 0.7 mL of RBC suspension or plasma was mixed with Ellman’s reagent containing 0.06 mM DTNB 0.2 M phosphate buffer pH 8.0 and incubated at room temperature for 50 min. Then, the OD was recorded at 420 nm using a UV–visible double-beam spectrophotometer. GSH concentration was determined from a standard curve of GSH standard (25–125 mg/mL). Quality control was indicated by a sensitivity of 0.26 mg GSH/mL, within a detection range of 0.26–122.8 mg GSH/mL, an intra-assay coefficient variation (CV) of 1.8%, and an inter-assay CV of 2.4%.

#### 2.8.7. Measurement of Lipid-Peroxidation Products

Malondialdehyde (MDA), as an index of lipid peroxidation, was determined from plasma samples using an HPLC-based thiobarbituric acid-reactive substance (TBARS) assay that had been previously described by Halliwell and Chirico [32]. In the assay, 27 µL of 5% RBC suspension (5% hematocrit or plasma was mixed with a reaction mixture containing 0.2% (*w*/*v*) BHT solution (3 µL), 0.44 M meta-phosphoric acid (180 µL), and 0.6% TBA (60 µL). The suspension was then incubated at 90 °C for 30 min and allowed to stand on ice for 10 min. Afterwards, the reaction mixture was treated with 150 µL of 20% (*w*/*v*) trichloroacetic acid, centrifuged at 12,000× *g* for 10 min, and the OD of the pink-colored product in the supernatant was photometrically measured at 532 nm against the reagent blank. TBARS concentration was determined from a standard curve of TMP and expressed as pmol/g Hb for RBC and µM for the plasma.

#### 2.8.8. Quantification of Non-Transferrin Bound Iron (NTBI)

Plasma NTBI was quantified based on the nitrilotriacetic acid (NTA) chelation/HPLC technique that had been previously described by Singh et al. with slight modifications [33]. Accordingly, 800 mM NTA pH 7.0 solution was prepared by mixing 800 mM disodium NTA with 800 mM trisodium NTA until the solution reached a final pH of 7.0. In our analysis, the plasma (450 μL) was incubated with 800 mM NTA pH 7.0 solution (50 μL) at room temperature for 30 min to transform NTBI to Fe-NTA. Afterwards, plasma proteins were removed by centrifugation of the treated plasma using an ultracentrifugation filtration device (NanoSep^®^, 30 kDa cut off, polysulfone type; Pall Life Sciences, Ann Arbor, MI, USA) at 12,000 rpm (10,620× *g*, Hettich Centrifugation, Hettich Lab Technology, Tuttlingen, Germany). This centrifugation process was done at 15 °C for 45 min. Ultrafiltrate was analyzed using a non-metallic HPLC system under the following conditions: a dual-piston high pressure pump (ConstaMetric^®^3500 LDC Analytical, Inc., Tampa, FL, USA), a glass analytical column (ChromSep ODS1, 100 × 10 mm, 5 μm; Chrompack International, Middelburg, The Netherlands), mobile-phase solvent containing 3 mM CP22 (1-ethyl-2-methyl-3-hydroxyl pyridinin-4-one) in 19% acetonitrile buffered with 5 mM MOPS pH 7.0 at a flow rate of 1.0 mL/minute. Column effluents were monitored at 450 nm using a flow-cell detector (SpecMonitor2300; LDC Milton-Roy Inc., Riviera Beach, FL, USA) and conducted with the BDS software version 1.2 (BarSpec Company Limited, Rehovot, Israel). The NTBI peak was calculated from a calibration curve of the standard iron solutions (0–16 μM Fe-NTA in 80 mM NTA pH 7.0). The NTBI concentration was determined from a calibration curve established from varied concentrations of 0–16 μM ferric nitrilotriacetate that had been prepared in 80 mM NTA pH 7.0.

### 2.9. Statistical Analysis

Statistical analyses were performed using IBM SPSS Statistics version 14 (IBM Corp., Armonk, NY, USA). Data are presented as mean ± standard deviation values (SD) for in vitro experiments and human RBC studies, whereas animal experimental data were expressed as mean ± standard errors of the mean values (SEM), unless otherwise indicated. Normality of data distribution was assessed prior to statistical comparisons. Comparisons between two groups were performed using Student’s *t*-test. For datasets with unequal variances, Welch’s *t*-test followed by Holm multiple-comparison correction was applied. Comparisons among multiple groups were analyzed using two-way analysis of variance (ANOVA) followed by the appropriate post hoc tests. Statistical significance was defined as *p* < 0.05. Sample sizes (n) represented independent biological replicates, individual subjects, or experimental animals as specified in each figure legend.

## 3. Results

### 3.1. Total Phenolic Content

The total phenolic contents (TPC) of GTE and BTE are summarized in Table 1. The results demonstrated that GTE possessed a higher total phenolic content than BTE, with values of 228.5 ± 82.6 mg GAE/g and 136.0 ± 31.9 mg GAE/g, respectively. The higher phenolic contents observed in GTE are consistent with the HPLC analysis presented in Section 3.1, which revealed higher concentrations of catechin derivatives, particularly EGCG, EGC, and EC.

### 3.2. Catechins and Caffeine Contents

**Figure 1:**
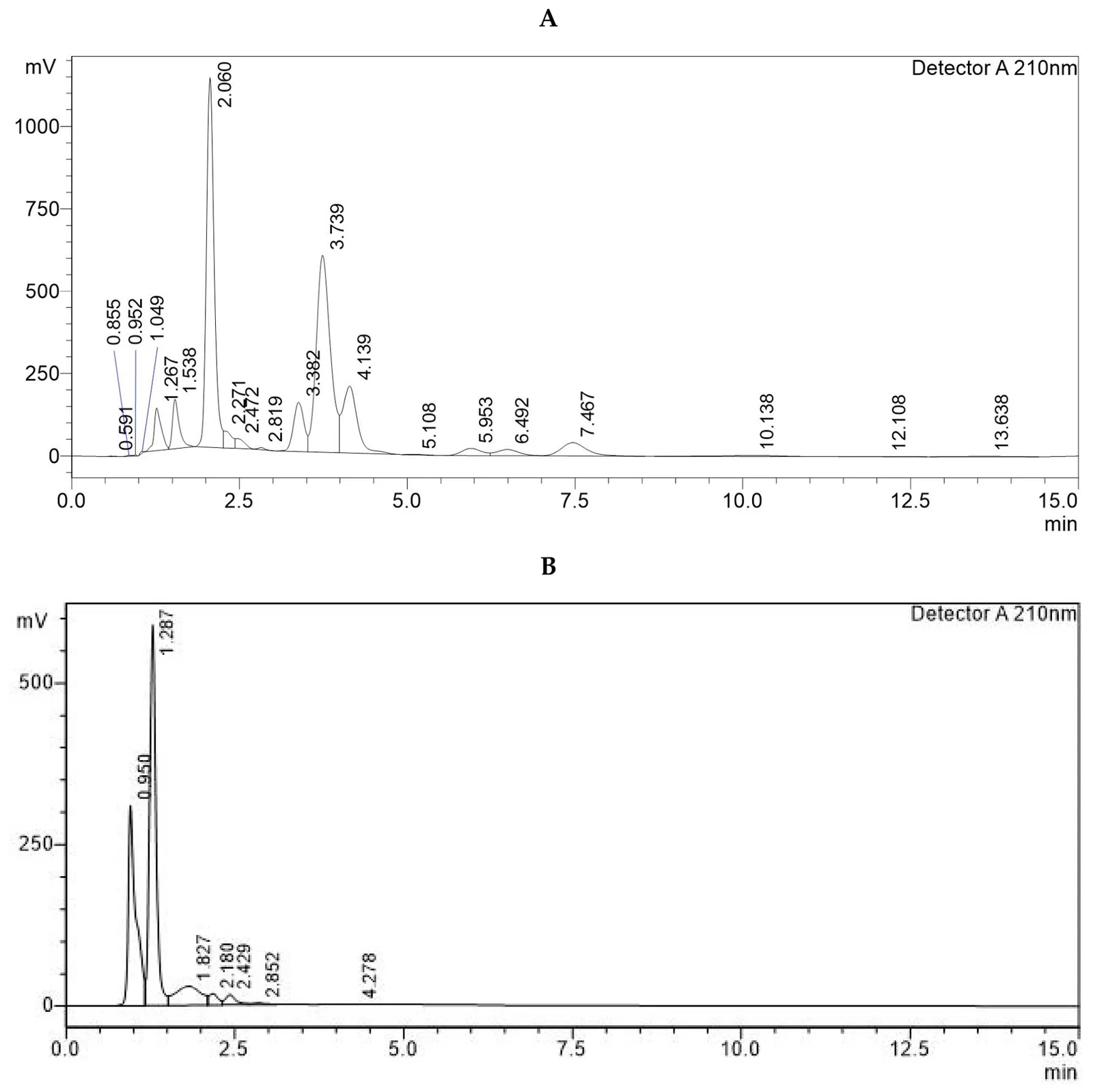
HPLC-PDA profile of catechin derivatives and CF in GTE (**A**) and BTE (**B**).

HPLC–PDA analysis demonstrated marked differences in phytochemical composition between GTE and BTE. EGCG was the predominant catechin in GTE, whereas several catechins were not detected in BTE (Figure 1). Overall, GTE contained higher concentrations of the quantified catechin derivatives than BTE (Table 2).

Among the detected compounds, EGCG was the predominant catechin in GTE, with a concentration value of 270.36 ± 0.75 mg/g, followed by CF (25.30 ± 0.07 mg/g), C (22.18 ± 1.25 mg/g), GCG (12.93 ± 0.63 mg/g), EGC (7.57 ± 0.01 mg/g), GA (7.34 ± 0.38 mg/g), and EC (6.37 ± 0.07 mg/g). In contrast, BTE contained markedly lower levels of catechin derivatives. GA was identified as the major detectable phenolic compound in BTE at 197.31 ± 0.20 mg/g, followed by C at 46.66 ± 0.25 mg/g, whereas CF was only detected at trace levels and several catechins, including EGCG, EC, EGC, and GCG, were not detectable.

These findings indicate that GTE possesses a richer catechin profile and higher antioxidant-associated phytochemical content than BTE, suggesting its greater potential for biological and functional applications.

### 3.3. Antioxidant Activity

**Figure 2:**
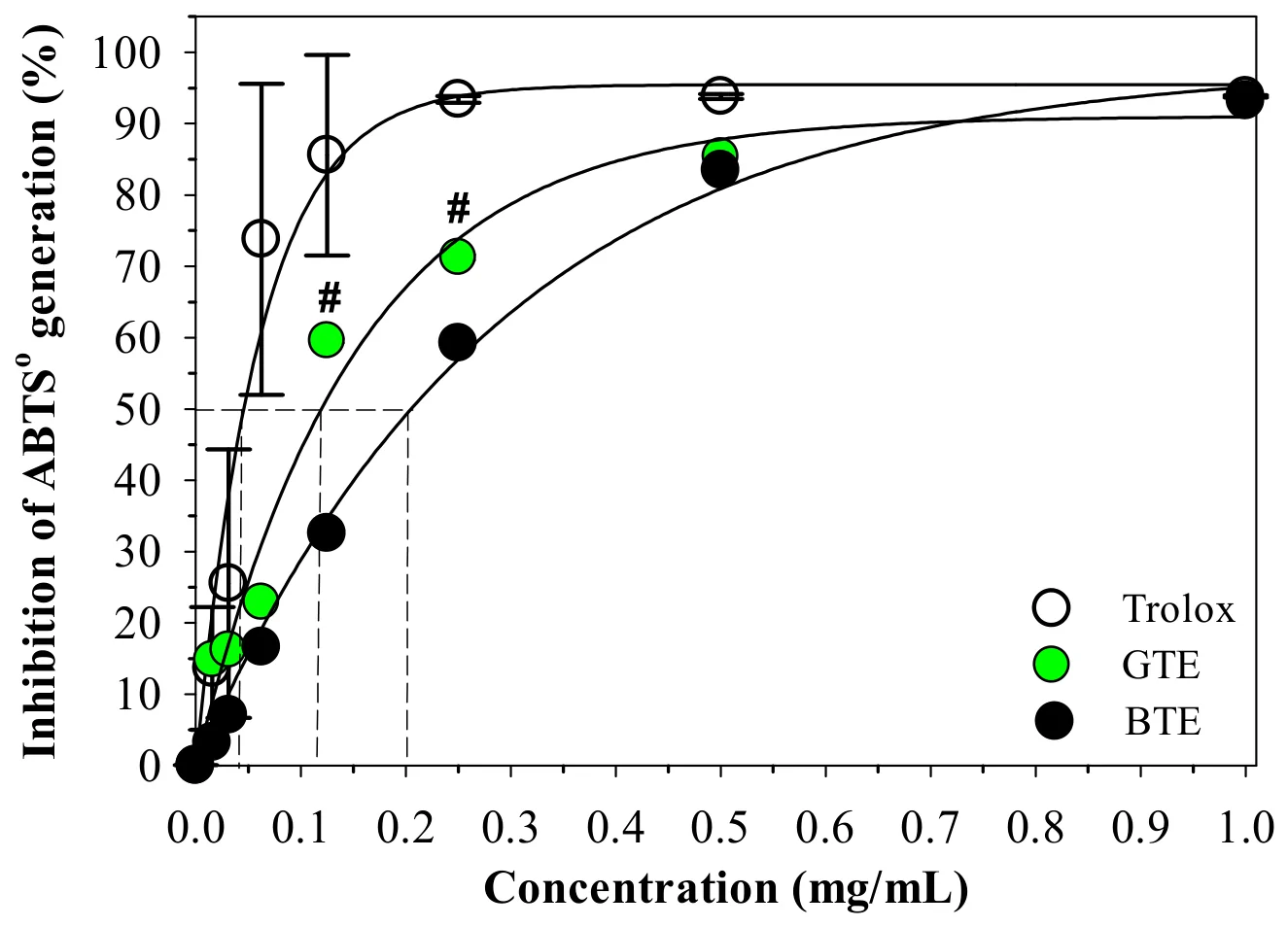
Antioxidant activity of GTE, BTE, and Trolox determined by the ABTS radical scavenging assay. Data are presented as the mean ± SD values obtained from three independent experiments (*n* = 3). Statistical comparisons between GTE and BTE at corresponding concentrations were performed using one-way ANOVA followed by post hoc multiple comparison testing. Accordingly, ^#^ *p* < 0.05 when compared with BTE.

The ABTS radical scavenging assay demonstrated that both GTE and BTE exhibited concentration-dependent antioxidant activity, with increasing concentrations resulting in higher degrees of inhibition of ABTS generation (Figure 2). However, GTE showed significantly stronger antioxidant activity than BTE at equivalent concentrations (0.1 and 0.2 mg/mL), as indicated by higher percentage inhibition values and statistical significance (^#^ *p* < 0.05). Trolox, used as the standard antioxidant reference, displayed the highest degree of activity at lower concentrations. Both extracts eventually reached high inhibition levels at higher concentrations.

In addition, IC_50_ values of ABTS^•+^ generation by Trolox, GTE, and BTE were found to be 0.047, 0.121, and 0.207 mg/mL, respectively, while the antioxidant activity values were 0.50 mg and 0.28 mg Trolox equivalent/g of the GTE and BTE, respectively.

### 3.4. Protective Effect of Tea Extracts Against RBC Hemolysis

#### 3.4.1. Normal and Thalassemia RBC

The healthy subjects and transfusion-dependent β-thalassemia patients included in this study were clinically characterized prior to oxidative hemolysis analysis. Demographic characteristics, diagnosis, splenectomy status, and iron-chelation therapy results are summarized in Supplementary Table S1, while hematological parameters are presented in Supplementary Table S2. Compared with normal controls, β-thalassemia subjects exhibited markedly lower Hb and hematocrit levels together with elevated red cell distribution width, which was consistent with chronic hemolytic anemia and the increased RBC fragility associated with β-thalassemia.

**Figure 3:**
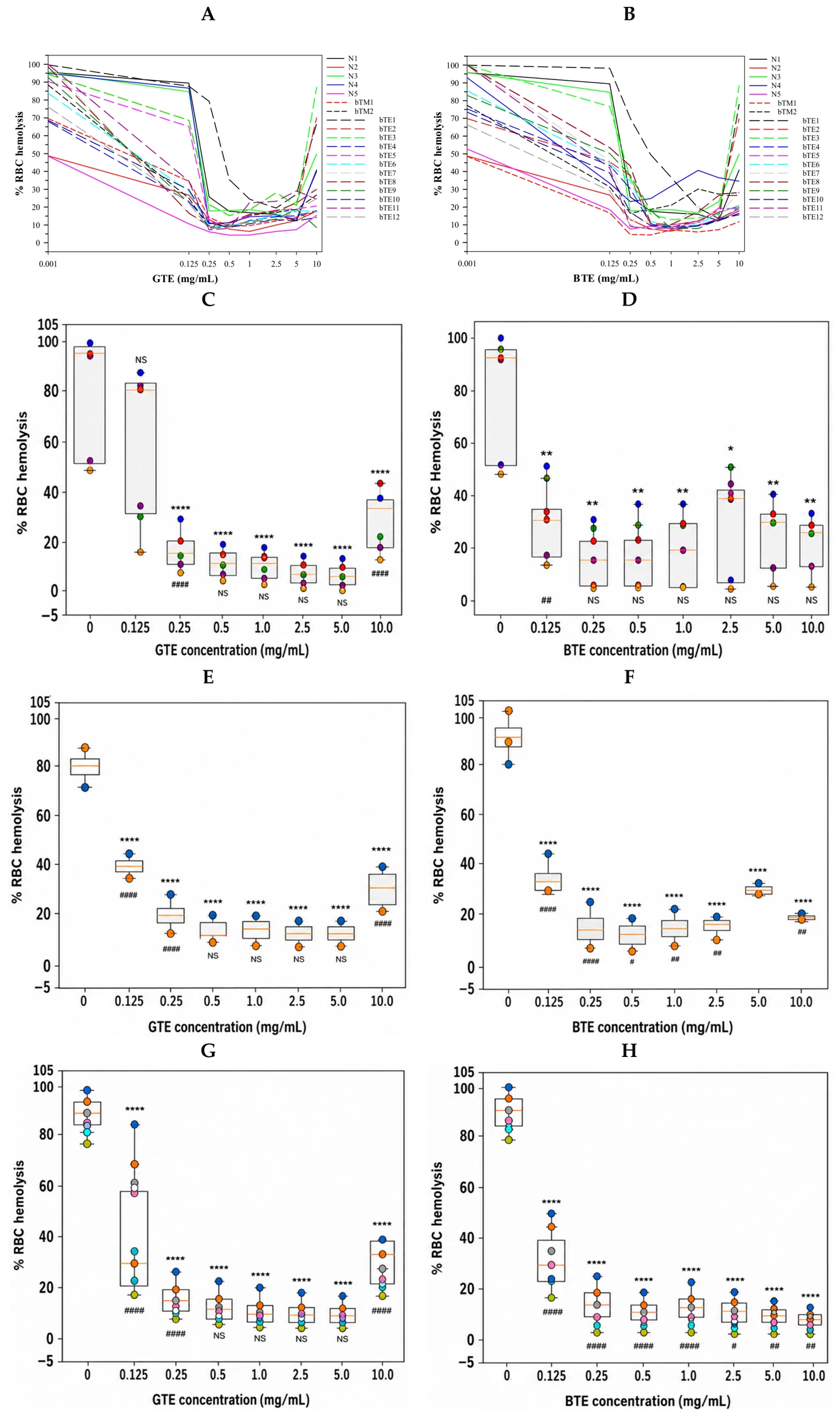
Effect of GTE and BTE treatment (0–10 mg/mL) on the hemolysis of normal and β-thalassemia RBC induced by AAPH. (**A**) Percentage hemolysis of normal and β-thalassemia RBCs treated with GTE; (**B**) percentage hemolysis of normal and β-thalassemia RBCs treated with BTE; (**C**) box-and-whisker plots showing individual measurements of normal RBCs treated with GTE; (**D**) normal RBCs treated with BTE; (**E**) β-thalassemia major RBCs treated with GTE; (**F**) β-thalassemia major RBCs treated with BTE; (**G**) β-thalassemia/HbE RBCs treated with GTE; and (**H**) β-thalassemia/HbE RBCs treated with BTE. Different colored dots represent individual biological samples (subjects) and are used solely to distinguish individual observations; the colors do not indicate additional experimental groups. Data are shown as box-and-whisker plots representing individual measurements obtained from independent experiments. Boxes indicate the interquartile range (IQR), center lines indicate medians, and whiskers represent minimum and maximum values. Statistical analyses were performed using Welch’s *t*-test followed by Holm multiple-comparison correction against the untreated control group (0 mg/mL). Significance levels were defined as * *p* < 0.05, ** *p* < 0.01, and **** *p* < 0.0001; NS = not significant.

As indicated by individual plots, both tea extracts possess antioxidant and cytoprotective properties capable of reducing oxidative hemolysis of RBC from normal subjects (*n* = 5) and β-thalassemia patients (*n* = 14) (Figure 3A). As shown in Figure 3B, both GTE and BTE significantly protected human RBC against AAPH-induced oxidative hemolysis in a concentration-dependent manner. At lower concentrations (e.g., 0.25 mg/mL), the protective effects of the two extracts were comparable. However, GTE generally produced greater reductions in hemolysis than BTE at intermediate and higher concentrations, which was consistent with its higher catechin content and stronger antioxidant activity that were indicated by the phytochemical analyses and ABTS assay. According to the box-and-whisker plots, GTE significantly protected RBC from normal subjects (*n* = 5) against AAPH-induced oxidative hemolysis in a concentration-dependent manner; consequently, hemolysis markedly decreased following the GTE treatment, with the strongest protective effects observed at intermediate concentrations (0.5–5.0 mg/mL) (Figure 3C). Statistical analysis also showed significant reductions in hemolysis when compared with the untreated control group, indicating potent antioxidant and membrane-protective activities. In contrast, BTE also reduced oxidative hemolysis but exhibited weaker and less consistent protective effects than GTE (Figure 3D), in which the lower anti-hemolytic activity of BTE was likely due to the reduced catechin content, which resulted from the oxidative fermentation that had occurred during black tea processing. Similarly, the treatment of RBC from bTM (*n* = 2) with GTE significantly reduced the percentage of hemolysis in a concentration-dependent manner, with the strongest protective effects observed at intermediate concentrations. The reductions in hemolysis were statistically significant when compared with the untreated control group, indicating that GTE effectively protected thalassemic RBC membranes against oxidative injury (Figure 3E). In contrast, BTE also decreased oxidative hemolysis but showed weaker and less consistent protective activity than GTE (Figure 3F). The untreated bTE RBC exhibited markedly high hemolysis percentages, reflecting their increased susceptibility to oxidative membrane damage (Figure 3G,H). Consistently, treatment of the RBC from bTE patients (*n* = 12) with GTE significantly reduced hemolysis in a concentration-dependent manner, with the greatest protective effects observed at intermediate concentrations. The box plots demonstrated lower median hemolysis values and reduced variability following GTE treatment, indicating consistent antioxidant protection across experiments. Statistical analyses revealed significant reductions in hemolysis when compared with the untreated control group. In contrast, BTE also reduced oxidative hemolysis but exhibited weaker and less consistent protective effects than GTE, with higher median hemolysis values and greater variability among all samples.

Statistical analysis using Welch’s *t*-test revealed that no significant differences were observed between GTE-bTM and GTE-N at any concentration range (0.001–10 mg/mL) (Table S3), indicating that incorporation of BTM did not substantially alter the biological activity of the native GTE extract. Similarly, no significant differences were observed between BTE-bTM and BTE-N, suggesting that BTM modification also had minimal impact on the membrane stabilization capacity of BTE formulations. Comparisons between the native extracts, BTE-N and GTE-N, likewise revealed no statistically significant differences, indicating that both extracts possess broadly similar anti-hemolytic properties. In contrast, comparisons involving the BTE-modified formulations revealed concentration-dependent effects. Significant differences were detected between GTE-bTE and GTE-N, as well as between BTE-bTE and BTE-N, specifically at the highest tested concentration of 10 mg/mL. A similar pattern was observed when comparing GTE-bTE with GTE-bTM and BTE-bTE with BTE-bTM, where statistically significant differences emerged only at 10 mg/mL. These findings suggest that the modified BTE formulations behave similarly to their corresponding native or BTM-modified extracts at low-to-moderate concentrations, but exhibit altered biological responses under high-dose conditions. Nevertheless, direct comparisons between BTE-derived and GTE-derived modified formulations (BTE-bTM vs. GTE-bTM and BTE-bTE vs. GTE-bTE) showed no significant differences across all concentrations, indicating that both extract systems displayed comparable membrane stabilization behavior following formulation modification. The data suggest that formulation-dependent effects become evident mainly at elevated concentrations, while lower concentrations maintain relatively consistent anti-hemolytic activity among the tested extracts and modified systems.

#### 3.4.2. Protective Effects of GTE Against Iron/H_2_O_2_-Induced Hemolysis of WT and BKO Mouse RBC

**Figure 4:**
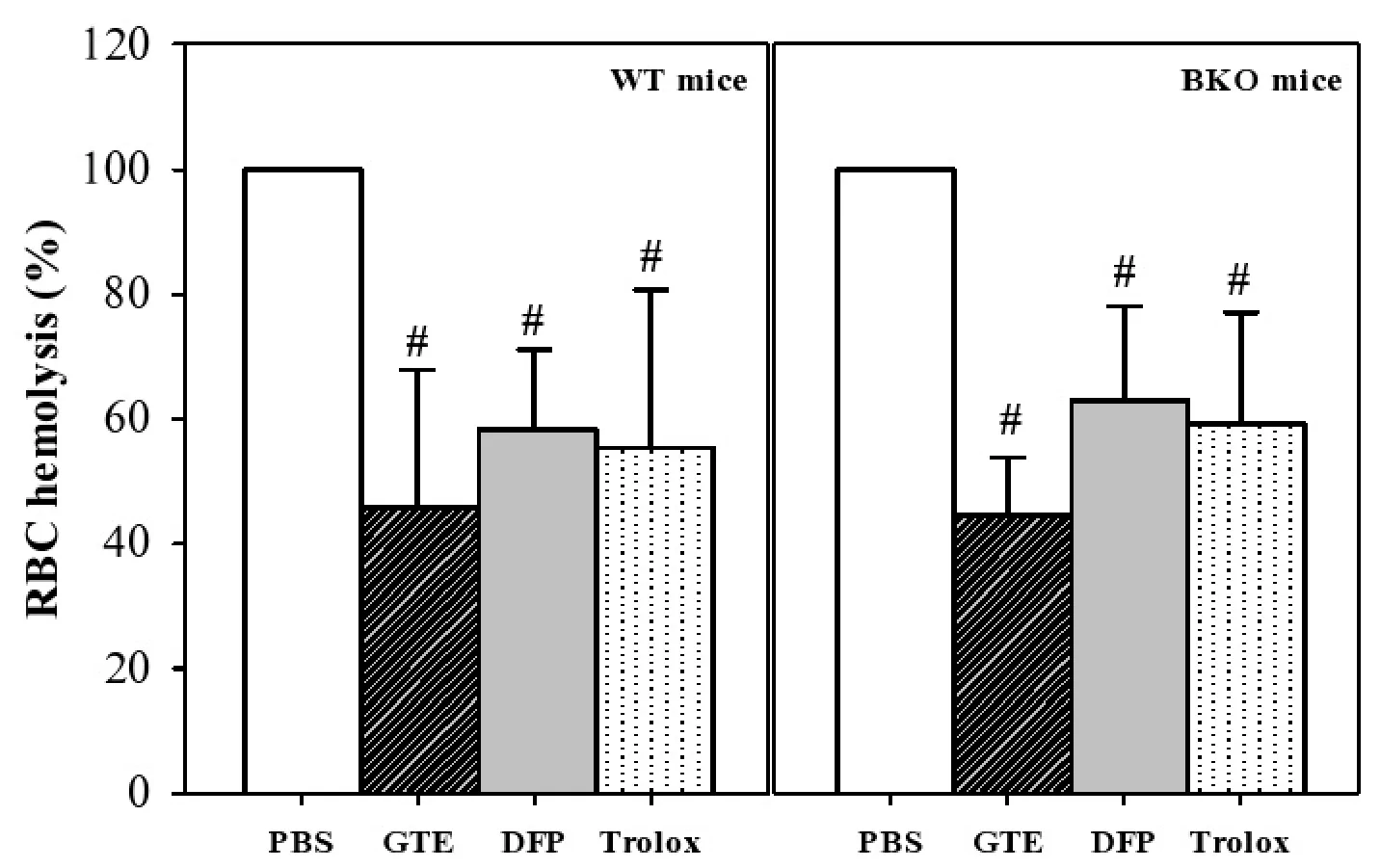
Percentage of hemolysis of WT and BKO RBC induced with FAS/H_2_O_2_ and treated with PBS, GTE (1.25 mg/mL), DFP (50 μM), and Trolox (50 μM). Data are expressed as mean ± SD values obtained from six independent experiments (*n* = 6). Statistical comparisons were performed using two-way ANOVA followed by Tukey’s post hoc test. ^#^ *p* < 0.05 compared with PBS-treated controls.

Figure 4 demonstrates the protective effect of GTE against oxidative hemolysis in WT and BKO RBC exposed to FAS/H_2_O_2_-induced oxidative stress. The figure indicates that treatment with GTE reduced the percentage of hemolysis in both WT and BKO RBC when compared with the PBS-treated control group, suggesting that GTE possesses antioxidant and cytoprotective properties capable of attenuating oxidative membrane damage. The antioxidant effect of GTE may be attributed to its high catechin and phenolic contents, particularly EGCG and the related polyphenols identified in the HPLC analysis.

The comparable protective effects of GTE with established antioxidant and iron-chelating agents further support its therapeutic potential. The statistically significant differences (^#^ *p* < 0.05) when compared with the PBS control indicate that the observed reductions in hemolysis were meaningful and not due to random variations. Overall, Figure 3 suggests that GTE effectively protects RBCs from oxidative hemolysis, especially under conditions associated with β-thalassemia-related oxidative stress, likely through combined antioxidant and metal-chelating mechanisms.

### 3.5. Effects of GTE Treatment on RBC and Oxidative Stress in Iron Overloaded Mice

**Figure 5:**
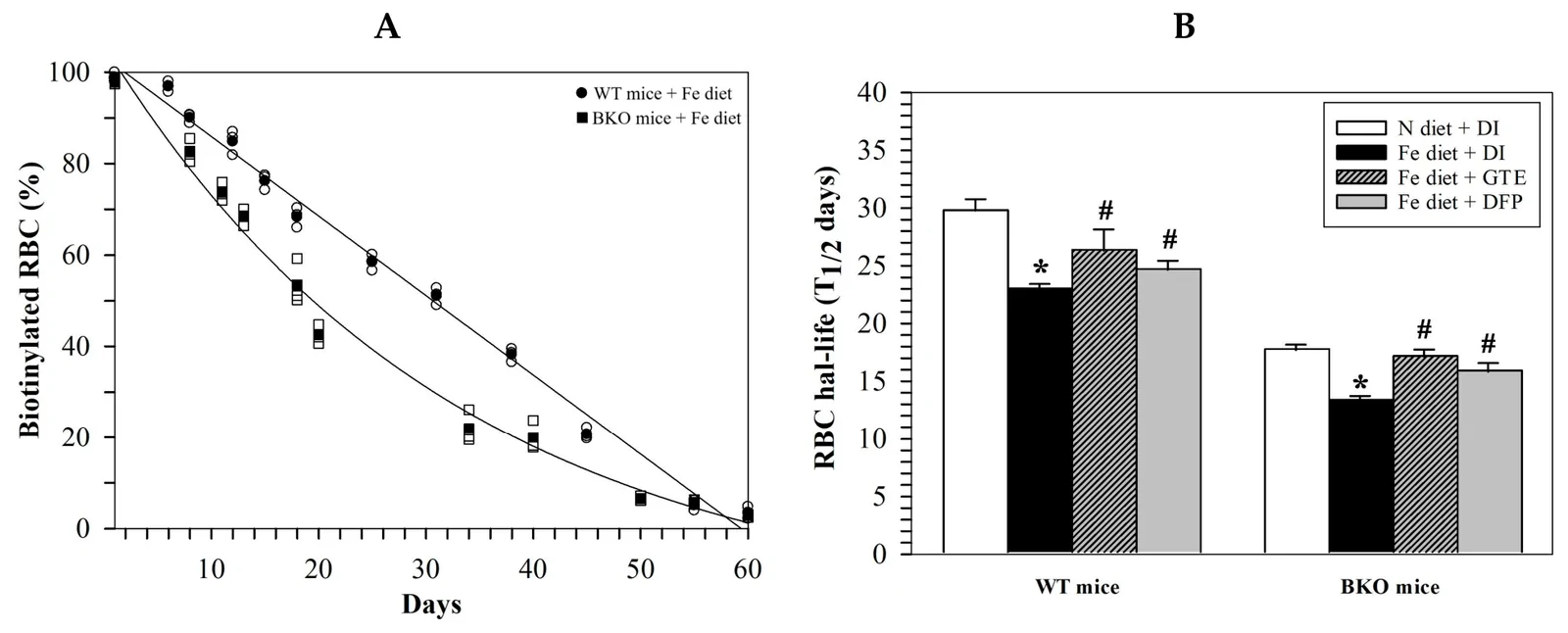
RBC survival values of individual WT and BKO mice (**A**) and mean RBC half-life values following treatment with DI, GTE (300 mg/kg), or DFP (50 mg/kg) for 2 months (**B**) Hollow circles and hollow squares represent individual WT and BKO mice, respectively. Data are presented as mean ± SD values (*n* = 3 mice/group). Statistical analyses were performed using two-way ANOVA followed by Tukey’s multiple-comparison post hoc test. * *p* < 0.05 versus N diet + DI group; ^#^ *p* < 0.05 versus Fe diet + DI group.

The results in Figure 5 demonstrate that iron overload adversely affected RBC survival, particularly in BKO mice, which are more vulnerable to oxidative stress and RBC membrane damage due to β-thalassemia-associated pathophysiology. Excess iron promotes ROS generation through Fenton-type reactions, leading to oxidative injury, membrane fragility, and shortened RBC lifespan. Treatment with GTE improved RBC survival in iron-overloaded mice when compared with the DI-treated iron overload group. This finding suggests that GTE exerts protective effects against oxidative RBC damage in vivo. The beneficial effects of GTE may be associated with its abundant catechin polyphenols, especially EGCG, which possess strong antioxidant and free radical scavenging activities. DFP, a clinically used iron-chelating agent, also improved RBC survival, supporting the role of iron-catalyzed ROS production in RBC destruction. The comparable degree of improvement observed with GTE suggests that tea polyphenols may contribute not only through antioxidant mechanisms but also potentially through mild iron-chelating activity. Therefore, chronic GTE administration can ameliorate the oxidative stress-related impairment of RBC survival in iron-overloaded β-thalassemic mice, highlighting its potential as a supportive natural antioxidant therapy for conditions associated with iron-induced oxidative damage.

**Figure 6:**
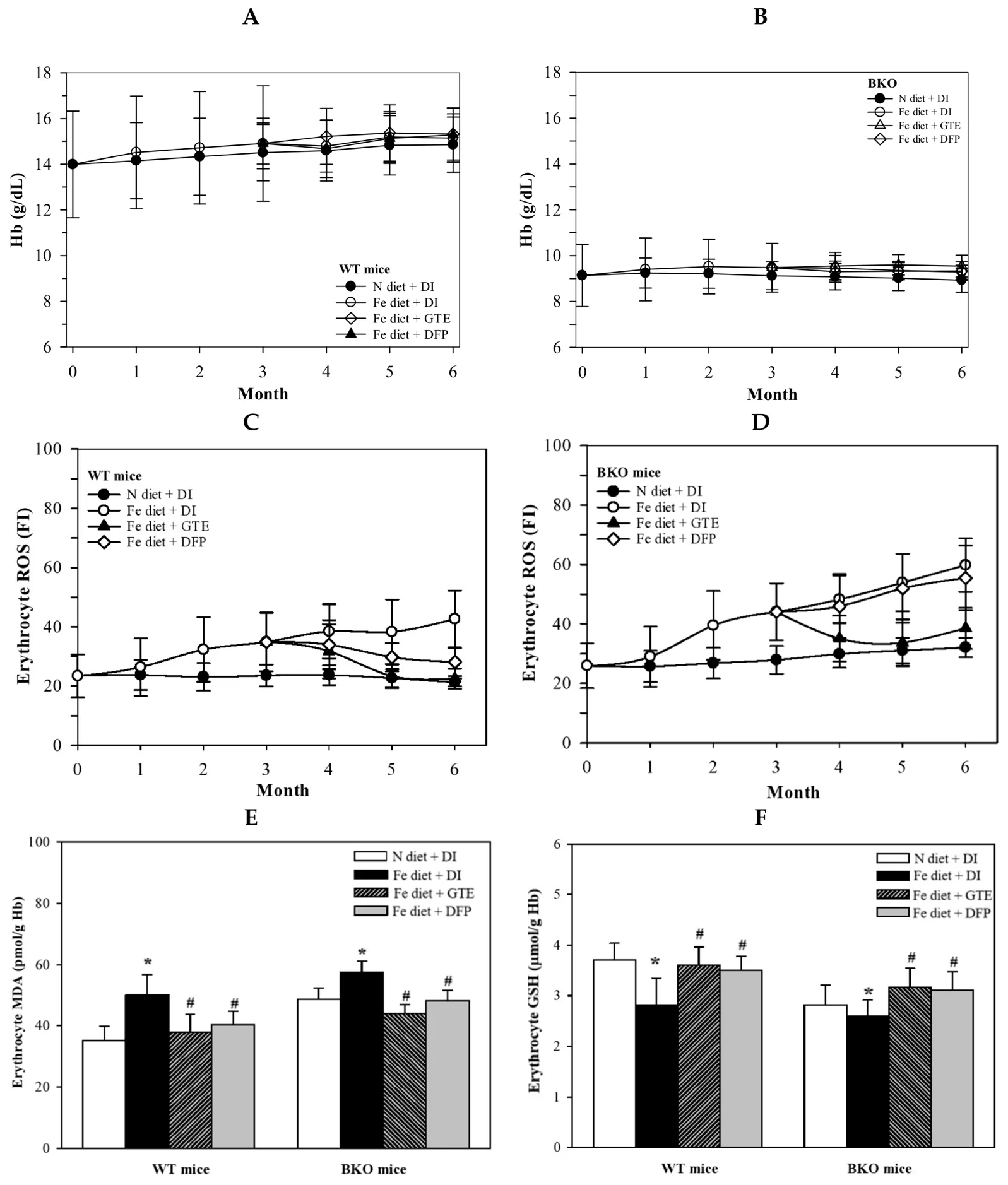
Effects of green tea extract (GTE) on hemoglobin (Hb) and red blood cell (RBC) oxidative stress in wild type (WT) and beta-globin knockout (BKO) mice subjected to chronic iron loading. (**A**,**B**) Hb concentrations monitored monthly in WT and BKO mice; (**C**,**D**) Intracellular reactive oxygen species (ROS) levels measured in erythrocytes during the experimental period; (**E**) RBC malondialdehyde (MDA) concentrations as an index of lipid peroxidation; (**F**) Intracellular glutathione (GSH) concentrations in erythrocytes. Mice were fed either a normal diet (N diet) or an iron-enriched diet (Fe diet) for six months and received deionized water (DI), GTE (300 mg/kg/day), or DFP (50 mg/kg/day) during months 3–6. Data are expressed as mean ± SD values (*n* = 12 mice/group). Statistical analyses were performed using two-way ANOVA followed by Tukey’s multiple-comparison post hoc test. * *p* < 0.05 versus N diet + DI group; ^#^ *p* < 0.05 versus Fe diet + DI group.

To evaluate whether chronic GTE supplementation alleviates oxidative damage associated with iron overload, hematological and oxidative stress parameters were monitored in WT and BKO mice during six months of dietary iron loading. As shown in Figure 6A,B, WT mice maintained Hb concentrations of approximately 14–15 g/dL throughout the experimental period regardless of dietary iron loading or treatment. In contrast, BKO mice consistently exhibited significantly lower Hb levels (approximately 9–10 g/dL), reflecting the chronic anemia characteristic of β-thalassemia. Neither GTE nor DFP markedly altered Hb concentrations in either genotype during the treatment period. Intracellular ROS levels increased progressively in erythrocytes from iron-overloaded mice (Figure 6C,D). The increase was particularly pronounced in BKO mice receiving the iron-enriched diet and vehicle treatment, indicating enhanced oxidative stress resulting from the combined effects of β-thalassemia and iron overload. Administration of GTE significantly suppressed erythrocyte ROS accumulation throughout the treatment period, producing reductions comparable to those observed with DFP. Consistent with the ROS findings, RBC MDA, a marker of lipid peroxidation, was significantly elevated following iron loading in both WT and BKO mice (Figure 6E). Baseline MDA levels were higher in BKO mice than WT mice, indicating greater susceptibility of thalassemic erythrocytes to oxidative membrane damage. Treatment with GTE significantly reduced MDA concentrations in iron-overloaded animals, restoring values toward those observed in mice receiving the normal diet. Similar protective effects were observed following DFP administration. Iron overload also reduced intracellular GSH concentrations (Figure 6F), indicating depletion of endogenous antioxidant defenses. GTE treatment significantly restored erythrocyte GSH levels in both WT and BKO mice, whereas DFP produced a comparable improvement. Restoration of intracellular GSH was accompanied by decreased ROS generation and lipid peroxidation, suggesting preservation of erythrocyte redox homeostasis. Collectively, these findings demonstrate that chronic GTE administration effectively attenuated oxidative stress in erythrocytes by reducing ROS production and membrane lipid peroxidation while restoring intracellular antioxidant capacity in iron-overloaded WT and β-thalassemic mice.

The relevant statistically significant differences (* *p* < 0.05 and ^#^ *p* < 0.05) indicate that the observed changes following GTE or DFP treatment were meaningful when compared with the control groups. Taken together, GTE can effectively mitigate oxidative stress in RBC by lowering ROS and lipid peroxidation while restoring antioxidant capacity, thereby protecting RBC from iron-induced oxidative damage.

**Figure 7:**
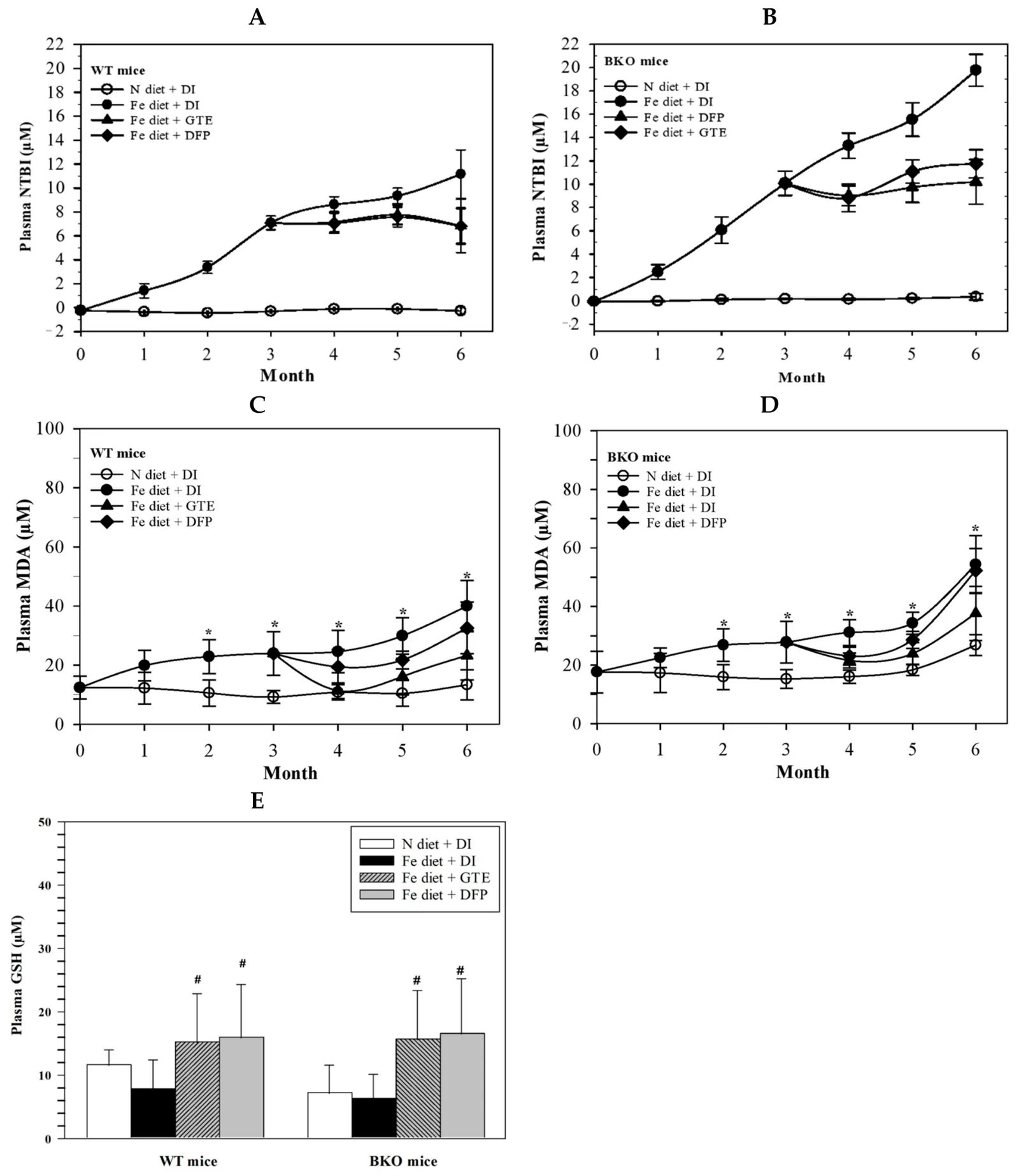
Plasma NTBI, MDA, and GSH levels in WT and BKO mice. (**A**) NTBI levels in WT mice; (**B**) NTBI levels in BKO mice; (**C**) MDA in WT mice; (**D**) MDA in BKO mice, and (**E**) GSH levels in WT and BKO mice. WT and BKO mice were fed either a normal diet (N diet) or an iron-enriched diet (Fe diet) for 6 months after being treated with DI, GTE (300 mg/kg), or DFP (50 mg/kg) during weeks 3–6. Data are expressed as mean ± SD values (*n* = 12 mice/group). Statistical analyses were performed using two-way ANOVA followed by Tukey’s multiple-comparison post hoc test. * *p* < 0.05 versus N diet + DI group; ^#^ *p* < 0.05 versus Fe diet + DI group.

WT and especially BKO mice receiving the Fe diet exhibited elevated plasma NTBI levels when compared with the normal diet control groups (Figure 7A). The marked increase in NTBI in BKO mice reflects the severe iron overload associated with β-thalassemia (Figure 7B). In addition, plasma MDA levels were also significantly increased in Fe diet-fed mice, particularly in the BKO animals (Figure 7C,D). Elevated MDA levels therefore confirm increased oxidative stress under iron overload conditions. Conversely, plasma GSH levels were decreased in the Fe diet groups, while treatment with GTE increased GSH concentrations when compared with the Fe diet + DI group (Figure 7E). These findings indicate that GTE effectively alleviated iron-induced oxidative stress and restored antioxidant capacity.

## 4. Discussion

The present study demonstrates that GTE effectively attenuates oxidative stress and improves RBC survival in iron-overloaded β-thalassemic models. Both in vitro and in vivo findings consistently showed that GTE reduced RBC hemolysis, lowered ROS, MDA, and plasma NTBI levels, while restoring GSH concentrations and prolonging RBC lifespan. These protective effects were observed in human β-thalassemia RBCs as well as BKO thalassemic mice, indicating that GTE exerts substantial cytoprotective and redox-regulating activities under iron overload conditions. The findings further support the concept that oxidative injuries play a central role in β-thalassemia-associated RBC instability and that the suppression of iron-dependent oxidative damage may improve RBC integrity and survival [3,12,15,16,34,35,36].

The HPLC-PDA analysis demonstrated that GTE contained substantially higher concentrations of catechin derivatives than BTE, particularly EGCG, which was identified as the predominant catechin compound. Consistent with this phytochemical profile, GTE exhibited greater total phenolic content and stronger ABTS radical scavenging activity than BTE. The outcomes of previous studies indicated that green tea processing preserves native catechin structures, whereas oxidative fermentation during black tea production converts catechins into polymerized compounds, such as theaflavins and thearubigins, which contribute to the characteristic color and flavor of black tea while retaining its antioxidant properties [37,38,39,40]. Catechin polyphenols, especially EGCG, possess strong free radical scavenging and metal-chelating properties that contribute to cellular protection against oxidative injury [19,25,41]. In addition, recent evidence suggests that EGCG can modulate ferroptosis-related pathways and improve cellular resistance against oxidative stress through the preservation of intracellular antioxidant systems [22,28]. The superior antioxidant capacity observed in GTE therefore likely reflects the preservation of these bioactive catechin compounds during green tea processing.

Although the HPLC analysis identified and quantified the major catechin constituents of GTE, the cumulative concentration of these compounds should not be interpreted as representing the total phenolic content. The apparent discrepancy between the total phenolic content determined by the Folin–Ciocalteu assay and the cumulative concentration of individual compounds quantified by HPLC should be interpreted in the context of the different analytical principles of the two methods. The Folin–Ciocalteu assay measures the overall reducing capacity of a broad spectrum of phenolic and other reducing substances and therefore estimates the total phenolic pool rather than that of individual compounds [20,42]. In contrast, the present HPLC method quantified only seven authentic reference compounds representing the major catechin constituents of GTE [14,43]. Consequently, numerous polymeric catechins, gallated derivatives, oxidized polyphenols, oligomeric flavan-3-ols, and other unidentified phenolic constituents were not included in the quantitative HPLC analysis. Therefore, the summed HPLC concentrations should not be expected to equal the total phenolic content obtained by the Folin–Ciocalteu assay [42].

Oxidative membrane injury is a major pathological feature of β-thalassemia and iron overload. Excess labile iron catalyzes ROS production through Fenton-type reactions, resulting in lipid peroxidation, membrane instability, and premature RBC destruction [13,15,16,36]. Persistent oxidative stress also contributes to ineffective erythropoiesis and shortened RBC lifespan. The use of two oxidative stress models was intentional because they represent different mechanisms of RBC oxidative injury. AAPH continuously generates peroxyl radicals and is commonly used to evaluate free radical-mediated membrane lipid peroxidation and antioxidant activity. In contrast, the iron/H_2_O_2_ system generates hydroxyl radicals via the Fenton reaction, closely mimicking the oxidative environment associated with iron overload in β-thalassemia patients. Since hydroxyl radicals are considerably more reactive than peroxyl radicals, a higher concentration of GTE was used in the iron/H_2_O_2_ experiments to provide sufficient antioxidant protection. Thus, the two models provide complementary information regarding the efficacy of GTE under distinct oxidative conditions. Thus, while BTE demonstrated antioxidant and cytoprotective activities, the higher catechin content of GTE likely contributed to its greater efficacy in reducing oxidative hemolysis and preserving RBC integrity.

The biological effects observed in the present study are likely attributable to the combined actions of multiple catechin derivatives identified in GTE, particularly EGCG, which represents the predominant constituent, together with C, GCG, EGC, EC, and GA. Following oral administration, catechins undergo intestinal absorption followed by extensive phase II metabolism, including glucuronidation, sulfation, and methylation, as well as enterohepatic circulation, resulting in relatively low systemic bioavailability but sustained biological activity through both circulating metabolites and local antioxidant effects [9,44,45,46]. Although plasma concentrations of intact EGCG are generally within the nanomolar-to-low micromolar range, repeated administration has been shown to maintain biologically relevant tissue exposure capable of modulating oxidative stress, iron metabolism, and intracellular antioxidant defense systems [44,46]. Experimental studies have demonstrated that EGCG directly scavenges ROS, chelates redox-active iron, activates the nuclear factor erythroid 2-related factor 2 antioxidant signaling pathway, suppresses nuclear factor kappa B -mediated inflammatory responses, preserves mitochondrial function, and inhibits lipid peroxidation and ferroptosis [11,47,48]. These complementary mechanisms provide plausible explanations for the reductions in ROS, MDA, and plasma NTBI together with restoration of GSH and prolonged RBC survival observed in the present study. Furthermore, the presence of multiple catechins and phenolic compounds may produce additive or synergistic biological effects that exceed those of individual purified compounds, supporting the therapeutic potential of whole GTE as a natural adjunctive intervention for β-thalassemia and other iron-overload disorders [11,45].

Despite these promising findings, the relatively low oral bioavailability of catechins remains an important consideration for clinical translation. EGCG and related catechins undergo extensive first-pass metabolism and exhibit considerable inter-individual variability in gastrointestinal absorption, plasma protein binding, and metabolic clearance, all of which may influence systemic exposure and therapeutic efficacy [44,46]. Nevertheless, repeated oral administration has consistently demonstrated biologically relevant antioxidant, anti-inflammatory, and iron-modulating effects in both experimental models and clinical studies [10,11]. Future investigations should therefore characterize the pharmacokinetic profiles of individual catechin metabolites, establish pharmacokinetic–pharmacodynamic relationships with biomarkers of oxidative stress and iron homeostasis, and evaluate novel delivery systems, such as nanoformulations, phospholipid complexes, or sustained-release formulations, to improve catechin stability, bioavailability, and therapeutic efficacy [45,46,49].

Herein, both human β-thalassemia RBC and BKO mouse RBC exhibited increased susceptibility to oxidative hemolysis. Treatment with GTE markedly reduced AAPH- and FAS/H_2_O_2_-induced hemolysis, indicating effective membrane protection against oxidative injuries. In iron-overloaded mice, chronic GTE administration also reduced ROS accumulation and MDA formation while restoring intracellular and plasma GSH levels toward normal values. The present findings are consistent with recent evidence showing that iron-associated oxidative stress in β-thalassemic erythrocytes promotes ROS accumulation, GSH depletion, lipid peroxidation, oxidation and redistribution of membrane proteins, cytoskeletal disruption, and impaired deformability [49,50]. By suppressing ROS and MDA accumulation while preserving GSH, GTE may maintain redox balance, reduce oxidative membrane remodeling and thereby delay premature erythrocyte clearance [3,12]. The observed improvement in RBC half-life following GTE treatment therefore likely results from combined suppression of oxidative membrane damage and enhancement of antioxidant defense capacity. Oxidative membrane remodeling can also increase erythrocyte recognition and removal by the reticuloendothelial system, thereby shortening RBC survival [18,29]. Restoration of redox homeostasis by GTE may therefore stabilize RBC membranes and reduce premature RBC removal.

The protective effects of GTE were comparable to those produced by DFP, a clinically established oral iron chelator [8,51]. Similar reductions in oxidative biomarkers and improvements in RBC survival were observed following treatment with either GTE or DFP, supporting the importance of iron-dependent oxidative injuries in β-thalassemia pathophysiology. In addition to its antioxidant activity, GTE significantly reduced plasma NTBI levels, suggesting that catechin polyphenols may also exert biologically relevant iron-interacting effects. The protective effects of GTE were comparable to those produced by DFP, a clinically established oral iron chelator [8,51]. Similar reductions in oxidative biomarkers and improvements in RBC survival were observed following treatment with either GTE or DFP, supporting the importance of iron-dependent oxidative injury in β-thalassemia pathophysiology. In addition to its antioxidant activity, GTE significantly reduced plasma NTBI levels, suggesting that catechin polyphenols may also exert biologically relevant iron-interacting effects. The reduction in plasma NTBI following GTE treatment is biologically relevant because NTBI comprises chemically heterogeneous, redox-active iron species that contribute to tissue iron uptake and iron-catalyzed oxidative injury [5,52]. Experimental evidence that EGCG can regulate iron metabolism and attenuate iron-induced oxidative injury supports the possibility that the catechin-rich extract exerted both radical-scavenging and iron-interacting effects [11]. Nevertheless, the present findings do not establish GTE as a substitute for clinically validated iron chelation. Basal NTBI levels were not higher in BKO mice. However, our results support the concept that oxidative stress is increased in BKO mice independently of basal NTBI, because ROS and MDA are elevated and GSH is depleted. Chronic dietary iron loading substantially increased erythrocyte oxidative stress, particularly in β-thalassemic mice. This was evidenced by progressive ROS accumulation, increased lipid peroxidation, and depletion of intracellular glutathione. These findings are consistent with the established pathophysiology of β-thalassemia, in which chronic iron overload and unstable globin chains generate excessive reactive oxygen species that accelerate erythrocyte membrane damage and premature cell destruction. Importantly, GTE markedly attenuated oxidative stress by suppressing ROS generation, reducing MDA formation, and restoring intracellular GSH. These protective effects were comparable to those produced by DFP, suggesting that the antioxidant activity of GTE complements its iron-chelating properties. Catechin polyphenols, particularly EGCG, have previously been shown to scavenge free radicals, chelate redox-active iron, inhibit lipid peroxidation, and activate endogenous antioxidant defense systems, thereby preserving erythrocyte membrane integrity. Interestingly, GTE produced little improvement in Hb concentration despite significantly reducing oxidative stress. This observation suggests that the principal action of GTE is preservation of erythrocyte survival rather than stimulation of erythropoiesis or correction of the underlying globin synthesis defect. Because β-thalassemia is caused by defective β-globin production, antioxidant therapy alone would not be expected to normalize Hb levels. Instead, reducing oxidative injury prolongs erythrocyte lifespan, thereby slowing hemolysis and improving erythrocyte quality. This interpretation is supported by the subsequent demonstration of prolonged RBC survival following GTE treatment. Overall, these findings indicate that GTE functions primarily as an antioxidant and membrane-protective agent that mitigates oxidative injury associated with iron overload rather than directly correcting anemia. Therefore, GTE may represent a promising adjunctive therapy to conventional iron chelation for reducing oxidative damage in β-thalassemia. Because long-term deferiprone therapy may be associated with adverse effects including gastrointestinal symptoms, arthropathy, neutropenia, and agranulocytosis [8,51], natural compounds possessing antioxidant and mild iron-chelating activities may provide additional therapeutic value when used alongside conventional chelation therapy. These findings therefore support previous investigations proposing green tea catechins as complementary nutraceutical agents for the management of iron overload and oxidative injury [19,25,41].

The prolongation of RBC survival observed following GTE administration is likely mediated through multiple complementary mechanisms. Iron overload in β-thalassemia promotes the accumulation of NTBI, which catalyzes Fenton reactions and generates highly reactive hydroxyl radicals. These reactive oxygen species induce the lipid peroxidation of erythrocyte membranes, oxidize membrane proteins, and deplete intracellular glutathione, thereby increasing membrane fragility and accelerating premature erythrocyte clearance. In addition, GTE significantly reduced intracellular ROS and plasma lipid peroxidation while restoring GSH concentrations. These changes collectively indicate the attenuation of iron-mediated oxidative injury. The high catechin content of GTE, particularly EGCG, likely contributes to these protective effects through both direct free-radical scavenging and iron-chelating activities, thereby interrupting the cycle of oxidative damage and preserving erythrocyte membrane integrity. Consequently, reduced oxidative hemolysis contributes to the prolonged RBC survival observed in iron-overloaded β-thalassemic mice.

The present study possesses several important strengths and potential benefits. First, the combination of ex vivo human RBC experiments and in vivo β-thalassemic mouse studies has provided a comprehensive evaluation of the protective effects of GTE under clinically relevant iron overload conditions. The use of both human β-thalassemia RBC and BKO mice enhanced the translational relevance of the findings and demonstrated consistent antioxidant and cytoprotective effects across multiple experimental systems. Second, the study comprehensively assessed several oxidative stress biomarkers, including ROS, MDA, GSH, plasma NTBI, RBC hemolysis, and RBC survival, thereby providing integrated insight into RBC redox homeostasis and membrane stability. Third, phytochemical characterization by HPLC-PDA confirmed high concentrations of catechin derivatives, particularly EGCG, supporting the mechanistic relationship between catechin composition and biological activity. Finally, a direct comparison with deferiprone strengthened the clinical significance of the findings and highlighted the potential adjunctive therapeutic value of GTE in iron-overload disorders [8,19,25,41].

Several translational considerations should be acknowledged. A limitation of this study is the relatively small number of healthy control subjects and the unequal sample sizes between the healthy and β-thalassemia groups. The smaller healthy cohort was intentionally used as a reference group because the primary objective was to investigate the effects of GTE in the target patient population. Nevertheless, larger cohorts with balanced sample sizes are warranted in future studies to further validate the anti-hemolytic efficacy of GTE across diverse populations. The present findings were derived from ex vivo human RBC experiments and an iron-overloaded β-thalassemic mouse model. The findings, therefore, may not fully reflect the complexity of this human disease. Moreover, the dose of GTE administered to mice may not directly correspond to achievable dietary intake levels in humans. The biological activity of tea extracts is also influenced by phytochemical composition, particularly catechin content, which may vary according to cultivar, processing methods, extraction procedures, and product standardization. Importantly, the beneficial antioxidant, anti-hemolytic, and RBC-protective effects of GTE suggest that it may have potential as an adjunctive intervention that complements treatment with clinically approved iron chelators, which remain the cornerstone of iron-overload management in patients with β-thalassemia. Whether such combination strategies provide additional clinical benefits beyond those achieved with conventional iron-chelation therapy alone remains to be established.

Several limitations should be considered. Although the BKO mouse model reproduces many features of human β-thalassemia intermedia, extrapolations of these findings to human clinical settings remain limited [24]. Only a single dosage of GTE was evaluated in vivo, precluding assessment of dose–response relationships. Furthermore, the study primarily focused on oxidative stress biomarkers and RBC survival and did not include comprehensive analyses of tissue iron accumulation, organ histopathology, inflammatory responses, or ferroptosis-associated molecular pathways. Additional mechanistic investigations examining antioxidant signaling pathways, iron transport proteins, and iron regulatory mechanisms would further clarify the cytoprotective effects of GTE. Long-term safety, pharmacokinetic interactions, potential interactions with conventional iron-chelation therapies, and the effects of chronic tea polyphenol consumption on mineral absorption were also beyond the scope of the present study. Finally, the relatively small sample size in some experimental groups, particularly in the β-thalassemia major subgroup, may have limited statistical power and generalizability. We acknowledge that the ex vivo evaluation was conducted using a relatively limited number of transfusion-dependent β-thalassemia patients. Therefore, these findings should be interpreted as supportive evidence of the biological activity of GTE and warrant validation in larger, well-powered clinical studies. Therefore, the present findings should be regarded as preclinical evidence supporting further mechanistic investigations and well-designed clinical studies to establish the efficacy, optimal dosage, safety, and therapeutic utility of GTE as an adjunct to standard iron-chelation therapy.

## 5. Conclusions

Both GTE and BTE demonstrated antioxidant and anti-hemolytic activities; however, GTE exhibited substantially greater efficacy, likely due to its higher catechin content, particularly EGCG and related polyphenols. GTE effectively reduced oxidative stress, lipid peroxidation, and NTBI levels while improving antioxidant status and prolonging RBC survival in iron-overloaded β-thalassemic mice. In addition, GTE provided marked protection against oxidative hemolysis in RBC obtained from healthy subjects and TDT patients. These findings highlight the importance of tea-derived polyphenols in mitigating oxidative injury associated with β-thalassemia and iron overload and provide additional evidence supporting the RBC-protective effects of GTE. Moreover, the findings are based on ex vivo human RBC experiments and a preclinical mouse model and should not be interpreted as evidence of clinical efficacy. Furthermore, GTE should be considered a potential adjunctive antioxidant approach that may complement, rather than replace, established iron-chelation therapies. Future studies are required to establish the safety, optimal dosing, standardization, long-term efficacy, and potential clinical benefits of GTE in β-thalassemia patients with and iron overload.

## Figures and Tables

**Table 1:** Total phenolic content of green tea extract and black tea extract. Data obtained from three independent triplicate experiments are expressed as mean ± SD values.

Analysis	GTE	BTE
TPC (mg GAE/g)	228.5 ± 82.6	136.0 ± 31.9

Abbreviations: BTE, black tea extract; GAE, gallic acid equivalent; GTE, green tea extract; TPC, total phenolic content.

**Table 2:** Catechin derivatives and CF present in GTE and BTE.

T_R_	GTE		BTE		Compound
(minute)	PA	(mg/g)	PA	(mg/g)	
0.591	6136	-	2,476,180	-	Unidentified
0.855	16,201	-	-	-	Unidentified
0.952	11,687	-	-	-	Unidentified
1.049	19,639	-	-	-	Unidentified
1.267–1.287	954,629	7.34 ± 0.38	4,148,422	197.31 ± 0.20	GA
1.538	1,067,821	12.93 ± 0.63	ND	ND	GCG
1.827	-	-	782,717	-	Unidentified
2.060	8,707,407	-	-	-	Unidentified
2.180–2.271	433,333	22.18 ± 1.25	167,550	46.66 ± 0.25	C
2.429–2.472	275,215	-	173,995	-	Unidentified
2.819–2.852	42,643	-	31,886	-	Unidentified
3.382	1,613,105	6.37 ± 0.07	ND	ND	EC
3.739	8,782,922	270.36 ± 0.75	ND	ND	EGCG
4.139–4.278	3,060,841	25.30 ± 0.07	3625	ND	CF
5.108	29,554	-	-	-	Unidentified
5.953	461,946	-	-	-	Unidentified
6.492	420,727	-	-	-	Unidentified
7.467	1,048,274	7.57 ± 0.01	ND	ND	EGC
10.138	207,737	-	-	-	Unidentified
12.108	29,140	-	-	-	Unidentified
13.638	58,420	-	-	-	Unidentified

Abbreviations: BTE = black tea extract, C = catechin, CF = caffeine, EC = epicatechin, EGC = epigallocatechin, EGCG = epigallocatechin-3-gallate, GA = gallic acid, GCG = gallocatechin-3-gallate, GTE = green tea extract, ND = not detectable, PA = peak area, T_R_ = retention time.

## Data Availability

Data are contained within the article or in the Supplementary Materials provided.

## Supplementary Materials

The following supporting information can be downloaded at: https://www.mdpi.com/article/10.3390/antiox15080990/s1, Table S1. Demographic data of normal and β-thalassemia subjects; Table S2. Hematological analysis of blood from normal and β-thalassemia subjects; and Table S3. Statistical comparison of RBC hemolysis responses among GTE- and BTE-derived formulations at equivalent concentrations.

## Abbreviations

The following abbreviations are used in this manuscript:

AA	Ascorbic acid
AAPH	2,2′-Azobis(2-amidinopropane) dihydrochloride
ABTS	2,2′-Azino-bis(3-ethylbenzothiazoline-6-sulfonic acid) diammonium salt
ANOVA	Analysis of variance
BHT	Butylated hydroxytoluene
BKO	β-Globin knockout
BTE	Black tea extract
bTE	β-Thalassemia hemoglobin E
bTM	β-Thalassemia major
C	Catechin
CF	Caffeine
CP22	1-Methyl-2-propyl-3-hydroxypyridine-4-one
CV	Coefficient of variation
DFP	Deferiprone
DI	Deionized water
DMSO	Dimethyl sulfoxide
DTNB	5,5-Dithio-bis (2-nitrobenzoic acid)
EC	Epicatechin
ECG	Epicatechin gallate
EGC	Epigallocatechin
EGCG	Epigallocatechin-3-gallate
FAC	Ferric ammonium citrate
FI	Fluorescence intensity
GA	Gallic acid
GAE	Gallic acid equivalent
GCG	Gallocatechin 3-gallate
GSH	Reduced glutathione
GTE	Green tea extract
HEPES	4-(2-Hydroxyethyl)-1-piperazineethanesulfonic acid)
HPLC-PDA	High-performance liquid chromatography coupled with photodiode array detection
IC_50_	Half-maximal inhibitory concentration
IQR	Interquartile range
LOD	Limits of detection
MDA	Malondialdehyde
NS	Not significant
NTA	Nitrilotriacetic acid
NTBI	Non-transferrin-bound iron
OD	Optical density
PA	Peak area
PBS	Phosphate-buffered saline
Q	Quercetin
QE	Quercetin equivalent
RBC	Red blood cells
REC	Research Ethics Committee
ROS	Reactive oxygen species
SD	Standard deviation
SEM	Standard error of the mean
T_1/2_	Half-time
TBA	2-Thiobarbituric acid
TDT	Transfusion-dependent β-thalassemia
TMP	1,1,3,3-Tetramethoxypropane
TPC	Total phenolic content
TBARS	Thiobarbituric acid-reactive substances
WT	Wild type
Trolox	6-Hydroxy-2,5,7,8-tetramethylchroman-2-carboxylic acid
