# Supplementary material for: Green Tea Extract Attenuates Oxidative Stress and Prolongs Red Blood Cell Survival in Iron-Overloaded β-Thalassemic Mice
Source: Antioxidants (Basel). 2026 Aug 10;15(8):990. doi: 10.3390/antiox15080990 (PMC13509167; doi:10.3390/antiox15080990)
Supplement: Supplementary file 1 [file antioxidants-15-00990-s001.zip › antioxidants-4367305-supplementary.pdf]

Supplementary Information

Table S1. Demographic data of normal and β-thalassemia subjects.

Subjects	Age (yr)	Gender	Diagnosis	Iron chelation	Splenectomy
N1	23	F	Normal	No	No
N2	23	F	Normal	No	No
N3	31	F	Normal	No	No
N4	31	F	Normal	No	No
N5	31	F	Normal	No	No
**Mean ± SD**	**27.80 ± 4.38**	**5F**	**5 Normal**	**None**	**None**
bTM1	40	F	β-thalassemia major	Combination	Yes
bTM2	28	M	β-thalassemia major	DFO	Yes
bTE1	43	F	β-thalassemia Hb E	No	Yes
bTE2	41	F	β-thalassemia Hb E	DFX	No
bTE3	43	M	β-thalassemia Hb E	DFP	No
bTE4	39	M	β-thalassemia Hb E	DFX	Yes
bTE5	19	F	β-thalassemia Hb E	DFP	No
bTE6	68	M	β-thalassemia Hb E	DFP	No
bTE7	68	F	β-thalassemia Hb E	Combination	No
bTE8	32	M	β-thalassemia Hb E	DFP	No
bTE9	37	F	β-thalassemia Hb E	Combination	Yes
bTE10	31	M	β-thalassemia Hb E	DFP	No
bTE11	58	F	β-thalassemia Hb E	DFX	No
bTE12	57	F	β-thalassemia Hb E	DFX	No
**Mean ± SD**	**43.14 ± 14.68**	**6M, 8F**	**2bTM, 12bTE**	**1None, 1DFO, 5DFP, 3DFX, 13 combinations**	**5Yes, 9No**

Abbreviation: bTE = β-thalassemia hemoglobin E, bTM = β-thalassemia major, DFO = desferrioxamine, DFP = deferiprone, DFX = deferasirox, F = female, M = male.

Table S2. Hematological analysis of blood from normal and β-thalassemia subjects.

Subject	Hb (g/dL)	Hct (%)	WBC ($x10^3$/μL)	Neu (%)	Lym (%)	Mon (%)	PLT ($x10^5$/μL)	MCV (fL)	MCH (pg/cell)	MCHC (g/dL)	RDW (%)
N1	13.5	41.7	4.70	51.0	41.0	8.0	2.40	NA	NA	NA	NA
N2	11.3	34.6	5.69	44.0	45.0	11.0	3.20	75.9	24.8	32.7	14.7
N3	11.9	35.8	5.60	52.0	41.6	6.4	1.49	90.4	29.0	32.1	11.8
N4	13.0	40.1	6.15	63.9	27.8	8.3	2.92	90.3	29.3	32.4	13.0
N5	16.3	51.1	9.80	50.9	44.9	4.2	2.39	40.8	13.0	31.9	12.3
**Mean**	**13.2**	**40.7**	**6.39**	**52.4**	**40.1**	**7.6**	**2.48**	**74.4**	**24.0**	**32.3**	**12.9**
**± SD**	**± 1.9**	**± 6.5**	**± 1.98**	**± 7.2**	**± 7.1**	**± 2.5**	**± 0.65**	**± 23.4**	**± 7.6**	**± 0.4**	**± 1.3**
bTM1	6.9	24.3	8.45	44.8	44.2	6.7	4.49	56.5	16.0	28.4	26.6
bTM2	8.6	26.2	11.86	45.9	42.0	9.6	6.31	78.9	25.9	32.8	23.2
**Mean**	**7.8**	**25.2**	**10.16**	**45.4**	**43.1**	**8.2**	**5.40**	**67.7**	**20.9**	**30.6**	**24.9**
**± SD**	**± 1.2**	**± 1.3**	**± 2.41**	**± 0.8**	**± 1.6**	**± 2.1**	**± 1.29**	**± 15.8**	**± 7.0**	**± 3.1**	**± 2.4**
bTE1	6.5	21.6	6.17	53.7	38.0	8.3	7.15	69.7	21.0	30.1	28.3
bTE2	7.4	24.9	6.56	71.5	22.7	4.6	1.72	58.5	17.4	29.7	29.6
bTE3	5.6	17.6	3.85	62.1	28.8	5.2	0.58	70.1	22.3	31.8	26.9
bTE4	5.7	19.1	6.89	30.8	53.8	14.4	4.91	67.3	20.1	29.8	31.7
bTE5	8.2	25.9	6.35	55.7	37.2	4.6	2.74	65.4	20.7	31.7	32.4
bTE6	6.5	22.7	3.86	63.5	29.8	6.7	1.80	54.4	15.6	28.6	27.2
bTE7	7.0	22.3	4.99	66.4	23.4	5.6	1.03	65.5	20.6	31.4	28.8
bTE8	6.0	19.6	4.15	51.8	43.4	3.6	0.93	69.0	21.1	30.6	27.5
bTE9	7.3	26.4	3.65	44.7	44.6	8.9	3.91	76.5	21.2	27.7	31.9
bTE10	8.9	28.5	11.13	49.6	42.2	5.6	1.89	67.4	21.0	31.2	26.7
bTE11	8.3	27.5	9.58	59.9	31.3	5.1	2.42	70.0	21.1	30.2	25.6
bTE12	8.0	24.9	4.40	43.7	50.7	3.6	1.70	68.4	22.0	32.1	27.5
**Mean**	**7.1**	**23.4**	**6.00**	**54.4**	**37.2**	**6.4**	**2.57**	**66.8**	**20.3**	**30.4**	**28.7**
**± SD**	**± 1.1**	**± 3.4**	**± 2.38**	**± 11.3**	**± 10.2**	**± 3.0**	**± 1.90**	**± 5.7**	**± 1.9**	**± 1.3**	**± 2.2**

Abbreviation: bTE = β-thalassemia hemoglobin E, bTM = β-thalassemia major, Hb = hemoglobin, Hct = hematocrit, Lym = lymphocyte, MCH = mean corpuscular hemoglobin, MCHC = mean corpuscular hemoglobin concentration, MCV = mean corpuscular volume, Mon = monocyte, Neu = neutrophil, PLT = platelet, RDW = red cell distribution width, WBC = white blood cell,

Table S3. Statistical comparison of RBC hemolysis responses among GTE- and BTE-derived formulations at equivalent concentrations. P-values obtained from Welch's t-test comparing N, bTM and bTE groups across concentrations ranging from 0.001 to 10 mg/mL. Statistical significance was defined as $p < 0.05$.

**Concentration (mg/mL)**	**GTE**			**BTE**		
	N vs bTM	N vs bTE	bTM vs bTE	N vs bTM	N vs bTE	bTM vs bTE
0	0.417	0.702	0.795	0.426	0.719	0.795
0.125	0.353	0.735	0.451	0.254	0.356	0.451
0.25	0.768	0.240	0.230	0.895	0.241	0.230
0.5	0.757	0.539	0.521	0.265	0.685	0.521
1	0.354	0.557	0.483	0.128	0.896	0.483
2.5	0.798	0.563	0.483	0.123	0.213	0.483
5	0.455	0.565	0.938	0.200	0.197	0.938
10	0.154	0.004	0.046	0.254	0.002	0.046
